# A Diagnostic Solution for Lupus Anticoagulant Testing in Patients Taking Direct Oral FXa Inhibitors Using DOAC Filter

**DOI:** 10.3389/fmed.2021.683357

**Published:** 2021-05-31

**Authors:** Carine Farkh, Syrine Ellouze, Louis Gounelle, Mama Sad Houari, Jérôme Duchemin, Valérie Proulle, Michaela Fontenay, Xavier Delavenne, Georges Jourdi

**Affiliations:** ^1^Service d'Hématologie Biologique, AP-HP, Center-Université de Paris, Hôpital Cochin, Paris, France; ^2^Institut Cochin, CNRS UMR8104, INSERM U1016, Université De Paris, Paris, France; ^3^Institut National de la Santé et de la Recherche Médicale U1059, Dysfonctions Vasculaires et de L'Hémostase, Université de Lyon, Saint-Étienne, France; ^4^Laboratoire de Pharmacologie, Toxicologie, Gaz du Sang, CHU de Saint-Etienne, Saint-Étienne, France; ^5^Université de Paris, Innovative Therapies in Haemostasis, Inserm UMR_S1140, Paris, France; ^6^Faculté de Pharmacie, Université de Montréal, Montreal, QC, Canada; ^7^Center de Recherche de L'Institut de Cardiologie de Montréal, Montreal, QC, Canada

**Keywords:** rivaroxaban, lupus anticoaglant, antiphospholipid antibodies, direct oral anitcoagulant, apixaban

## Abstract

**Background:** Direct oral factor Xa (FXa) inhibitors interfere with lupus anticoagulant (LA) assays challenging antiphospholipid syndrome diagnosis in treated patients. We evaluated a new device, called DOAC Filter, and its usefulness in this setting. It is a single-use filtration cartridge in which FXa inhibitor compounds are trapped by non-covalent binding while plasma is filtered through a solid phase. Patient samples were analyzed before and after filtration: 38 rivaroxaban, 41 apixaban, and 68 none. Anticoagulant plasma concentrations were measured using specific anti-Xa assays and HPLC-MS/MS. LA testing was performed using dilute Russell Viper Venom Time (dRVVT) and Silica Clotting Time (SCT). Baseline median [min–max] concentrations were 64.8 [17.6; 311.4] for rivaroxaban and 92.1 ng/mL [37.1; 390.7] for apixaban (HPLC-MS/MS). They were significantly correlated with anti-Xa assay results (*r* = 0.98 and *r* = 0.94, respectively). dRVVT was positive in 92% rivaroxaban and 72% apixaban and SCT in 28 and 41% of samples, respectively. Post-filtration, median % of neutralization was 100% with rivaroxaban and apixaban concentrations of, respectively, <2 [<2–2.4] and <2 ng/mL [<2–9.6] using HPLC-MS/MS. No significant effect of DOAC Filter was observed on LA testing in controls (*n* = 31) and LA-positive (*n* = 37) non-anticoagulated samples. dRVVT and SCT remained positive in, respectively, 16 and 8% of rivaroxaban and 41 and 18% of apixaban samples. DOAC Filter would be an easy-to-use device allowing FXa inhibitor removal from plasma samples, limiting their interference with LA testing in treated patients.

## Introduction

Antiphospholipid syndrome (APS) is an immune disorder characterized by the association of at least one thromboembolic event and/or obstetrical complication (fetal death, miscarriages) with positivity of at least one persistent (≥ 12 weeks' interval) antiphospholipid antibody (aPL). These include lupus anticoagulant (LA), anti-cardiolipin (aCL), and anti-beta2-glycoprotein I (aβ_2_-GPI) antibodies (1, 2). A reliable diagnosis of APS is crucial to allow adequate therapeutic management. Indeed, given the high risk of recurrent thrombotic event in APS patients, particularly those with a first unprovoked event (3), aPL results might affect the choice of the anticoagulant drug as well as the treatment duration (4–7). The International Society on Thrombosis and Haemostasis (ISTH), the European Medicines Agency (EMA), the European League Against Rheumatism (EULAR), and the British Society of Hematology (BSH) did not recommend the use of FXa inhibitors in APS patients, especially those with triple positive aPL (8–11). ISTH, EULAR, and BSH recommended against their use also in APS patients with a history of arterial thrombosis (8, 9). The European Society of Cardiology recommended against their use in all APS patients (12). Nevertheless, FXa inhibitors are increasingly used to treat patients with thromboembolism, and it is far from uncommon to be prescribed prior to aPL diagnosis. Hence, accurate aPL testing is mandatory to avoid inappropriate use of these anticoagulant compounds in this population (4, 5). While detection of solid-phase antibodies aCL and aβ_2_-GPI is not affected by FXa inhibitor compounds present in tested samples (13, 14), it is well-established that rivaroxaban and apixaban have the potential to compromise LA testing leading to unreliable results (false-positive/negative results) even at very low concentrations (15–22). Due to their heterogeneity, LA testing should be performed using, at least, two coagulation assays of differing analytical principles, the first based on dilute Russell Viper Venom Time (dRVVT) and the second derived from activated partial thromboplastin time (aPTT) (6, 18, 23). Both assays are compromised by the presence of FXa inhibitor compounds in tested samples. Many options for their *in vitro* neutralization using specific antidotes (namely, idarucizumab and andexanet-alfa) (14, 18) or adsorption products such as activated charcoal (DOAC Stop™, DOAC Remove®) have been proposed to overcome their interferences with LA testing (18, 24–28). While specific antidotes would be an expensive solution and might lack availability, further investigation of the commercially available adsorption products is needed to prove the complete neutralization of apixaban and rivaroxaban in tested samples. As such, LA testing remains challenging in patients receiving FXa inhibitors owing to the absence of clear guidance and of an easy-to-use device allowing their complete *in vitro* removal from patients' samples.

Blood sample filtration using a new device, called DOAC Filter, has recently been studied (29) in spiked plasma samples. It is a single-use filtration cartridge in which FXa inhibitor compounds are trapped by non-covalent binding while plasma is filtered through a solid phase. We hence sought to evaluate its potential usefulness for LA testing in real-life clinical practice.

## Materials and Methods

### Plasma Samples

This non-interventional study was conducted at Cochin University Hospital (AP-HP. Center, Paris, France) in accordance with the ethical principles of the Declaration of Helsinki. Overall, 147 blood samples were collected into 0.109 M of buffered trisodium citrate (9:1 v/v) tubes (Greiner Bio One, Courtaboeuf, France) and referred to our hematology laboratory for LA testing following an episode of thromboembolic event. Patients gave their written informed consent allowing usage of the residual samples for research purposes. The study was conducted using unidentified samples. Seventy-nine samples were from patients receiving direct oral FXa inhibitors (41 apixaban and 38 rivaroxaban) and 68 from patients not receiving any anticoagulant therapy. Thirty-one out of these 68 samples were from patients known as LA negative and were used as the control group whereas 37 were from patients having positive dRVVT and/or Silica Clotting Time (SCT) and were used as the LA-positive group. Blood samples were double centrifuged as recommended (13) at 2,500 g for 15 min at room temperature with plasma decantation in a second tube in between, leading to platelet-poor plasma (PPP, i.e., <10 000 platelets/mL) which was frozen at −80°C until use. Just prior to experiment, PPP was thawed at 37°C, gently mixed, then tested within 2 h.

### dRVVT and SCT Assays

dRVVT and SCT are integrated assays performed on PPP samples using Sysmex CS 5100 (Siemens Diagnostics, Saint-Denis, France). LAC screening® and confirmation® (Siemens Diagnostics) were used for dRVVT assay. A protease extracted from the venom of the *Daboia russelii* viper directly activates the endogenous FX in the presence of calcium ions and phospholipids added at low (screen assay) or high (confirm assay) concentration resulting in a fibrin clot. SCT assays were performed using the SCT Hemosil® Silica Clotting Time Screen/Confirm reagents (Werfen, Le Pré-Saint-Gervais, France) in which colloidal silica activates the contact pathway coagulation factors in the presence of calcium ions and phospholipids added at low or high concentrations, respectively. In dRVVT and SCT assays, fibrin clot formation was detected optically. Hemosil® Normal control assayed (Werfen) was used as reference plasma and run in each series. Screened and confirmed results were reported as ratios of patients to reference plasma clotting time in order to mitigate issues related to analytical variability. Confirm assay was realized when the screen ratio was equal or above the cutoff value. The final result was expressed as a normalized ratio corresponding to the screen over the confirm ratios as recommended by the BSH (30). The cutoff value was 1.20 for both screen and screen/confirm ratios for both dRVVT and SCT assays as stated by the manufacturers and locally validated (16). The intra- and interassay coefficients of variation (CV) were 0.42 and 2.10% for dRVVT and 1.11 and 2.47% for SCT assays, respectively.

### Direct Oral FXa Inhibitor Concentration Measurement

Direct oral FXa inhibitor concentrations were measured in pre- and post-filtration plasma samples using drug-calibrated anti-Xa assays (STA Liquid Anti Xa, Stago) and/or a validated high-performance liquid chromatography coupled with electrospray ionization tandem mass spectrometry (HPLC MS/MS). The lower limits of quantification (LLOQ) of both assays were locally determined and were equal to 18 and 2 ng/mL, respectively.

### Samples Treatment With DOAC Filter

Treatment with DOAC Filter was performed according to the manufacturers' instructions. Briefly, the device is divided into three pieces: a cartridge containing the solid chemical phase (i.e., filter), the connector, and the STA® microtainer. Once the three pieces are connected together, 600 μL of PPP was loaded into the cartridge before centrifugation at 300 *g* during 15 min at room temperature. The STA® microtainer containing the post-filtration plasma was afterward loaded into the Sysmex CS 5100 in order to proceed with LA testing. One DOAC Filter was used per each patient sample.

### Study Design

dRVVT and SCT screen assays were performed before and after treatment with DOAC Filter in all patients' samples. No additional assay was performed in samples tested negative, while those having an elevated screen ratio (i.e., ≥ 1.20) were subsequently analyzed using confirm assays. FXa inhibitor plasma concentrations were measured using both specific anti-Xa assays and HPLC-MS/MS. The same samples were used for all steps of pre- and post-filtration testing.

### Data Analysis

FXa inhibitor plasma concentrations and the % of neutralization were expressed as median [min–max]. One hundred % of neutralization corresponds to the decrease in plasma concentration below the LLOQ of HPLC-MS/MS (i.e., <2 ng/mL). Correlations between FXa inhibitor concentrations measured with both methods and between anticoagulant plasma concentrations and LA results in pre-filtration samples were evaluated using the Spearman rank correlation. According to the distribution of the continuous variables (D'Agostino & Pearson normality test), screen and screen/confirm ratios were compared before and after sample treatment with DOAC Filter using the two-tailed paired *t-*test or Wilcoxon matched-pair signed-rank test. A *p* < 0.05 was considered as statistically significant. All statistical analyses and graph representation were computed using the GraphPad Prism 9.0.0 software (GraphPad, San Diego, CA).

## Results

### Neutrality of DOAC Filter With Regard to LA Assays in the Absence of Direct Oral FXa Inhibitors

**Figure 1:**
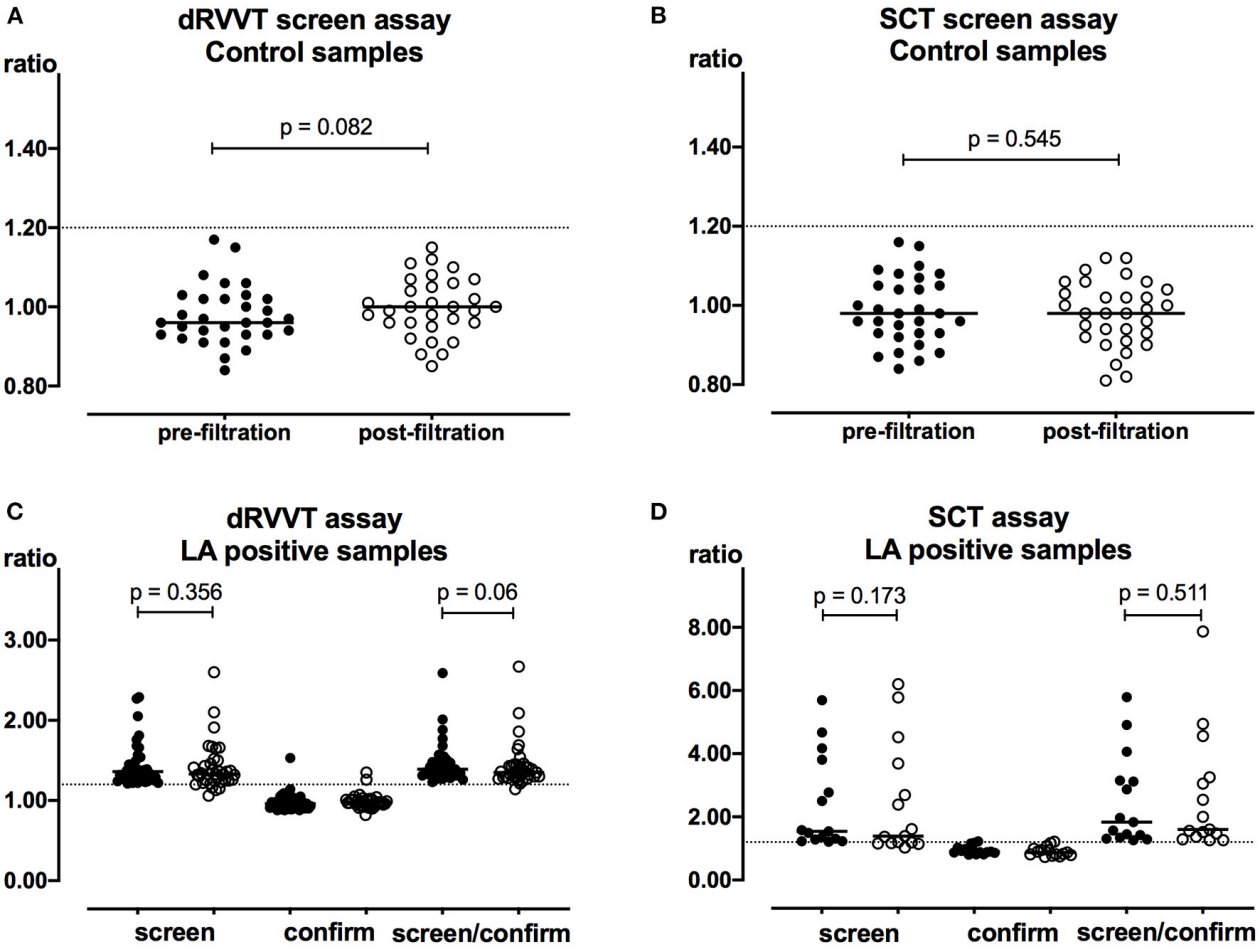
Neutrality of DOAC Filter with regard to LA assays in the absence of FXa inhibitors. Pre- (closed symbols) and post-filtration (open symbols) samples were tested using dRVVT **(A)** and SCT **(B)** screen assays in control samples (*n* = 31) and using dRVVT **(C)** and SCT **(D)** screen and confirm assays in LA-positive samples (*n* = 37 and 15, respectively). Dashed line corresponds to the cutoff value of 1.20. Horizontal lines represent median values.

Volume assessment was performed in a subset of 49 plasma post-filtration samples (26 control, four LA positive, three apixaban, and 16 rivaroxaban samples). The mean volume recovered was 420 μL (95% confidence interval (CI) [413–422]), corresponding to a mean plasma recovery of 70% with an inter-assay CV of 3.9%. Thirty-one samples from non-anticoagulated patients were tested LA negative using dRVVT and SCT screen assays and were used as controls. DOAC Filter did not affect dRVVT (*p* = 0.082) and SCT (*p* = 0.545) results (Figures 1A,B). Moreover, 37 samples from non-anticoagulated patients were tested positive with dRVVT assays. Fifteen out of 37 were also LA positive using SCT assays. DOAC Filter did not affect dRVVT screen (*p* = 0.356) or screen/confirm ratios (*p* = 0.06) (Figure 1C). It neither did with SCT screen (*p* = 0.173) or screen/confirm ratios (*p* = 0.511) (Figure 1D). However, despite no significant difference observed in the results between pre- and post-filtration control and LA-positive non-anticoagulated samples, four dRVVT and five SCT weakly elevated screen ratios turned out to be negative (i.e., <1.20) following treatment with DOAC Filter. Among these samples, only one DRVVT screen/confirm ratio turned out to be negative. The others remained positive. Results of these samples are detailed in Supplementary Table 1.

### Impact of Direct Oral FXa Inhibitors on LA Assays

**Figure 2:**
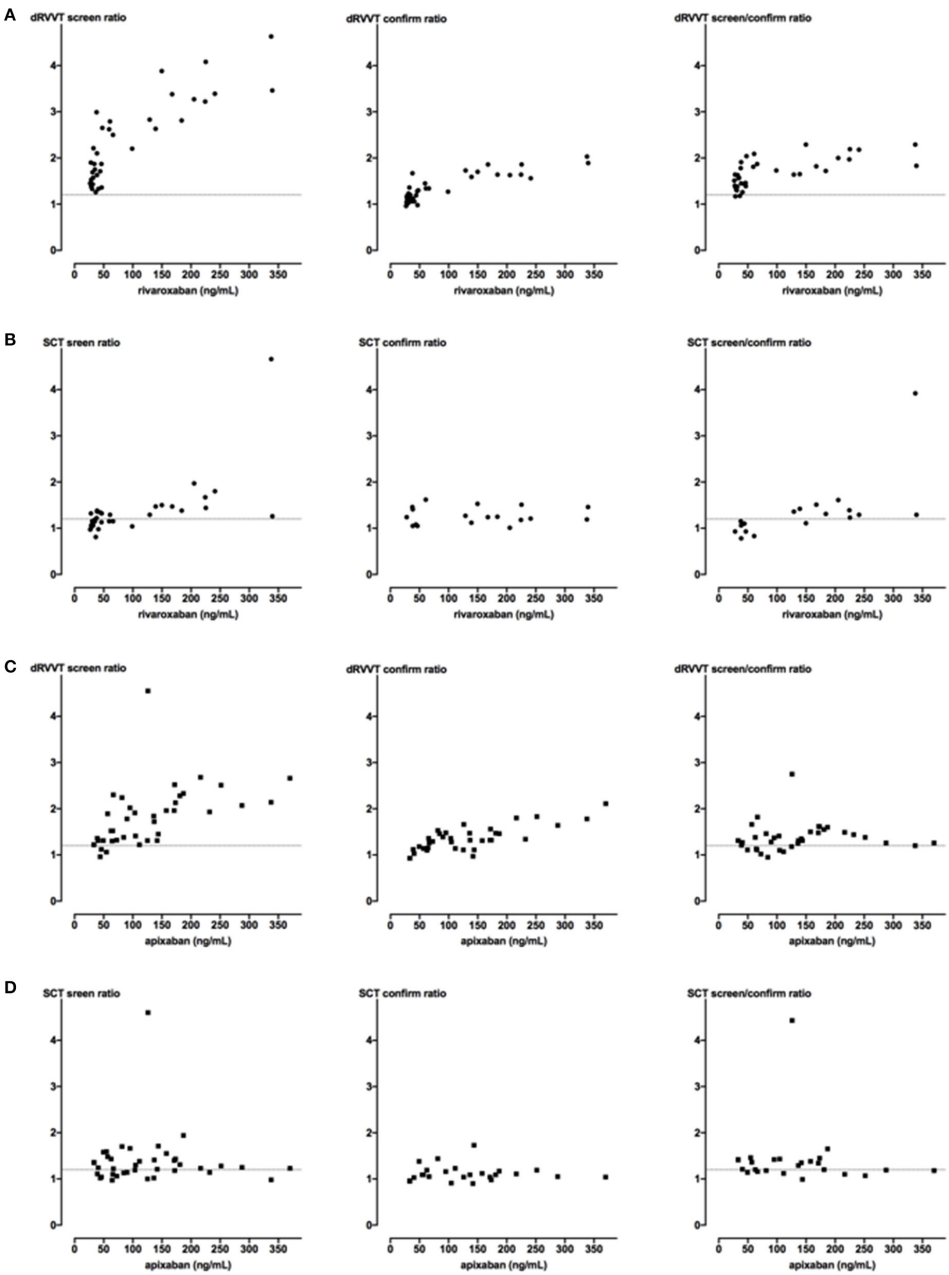
Impact of FXa inhibitors on dRVVT and SCT assays. Samples from patients receiving direct oral FXa inhibitors were tested using dRVVT (*n* = 38 in A and *n* = 39 in C) and SCT (*n* = 36 in B and *n* = 39 in D) screen assays. Only samples having elevated screen ratio (i.e., ≥ 1.20) were subsequently analyzed using confirm assays. Screen and confirm ratios are plotted as a function of rivaroxaban **(A,B)** or apixaban **(C,D)** plasma concentrations measured using specific anti-Xa assays. Dashed line corresponds to the cutoff value of 1.20.

LA testing was performed in plasma samples from 79 patients: 41 receiving apixaban and 38 rivaroxaban. FXa inhibitor plasma concentrations ranged from 17.6 to 311.4 ng/mL for rivaroxaban and 37.1–390.7 ng/mL for apixaban, as assessed by HPLC-MS/MS (Table 1). They were also measured using the widespread available methods, namely, specific anti-Xa assays (Table 1). The results of the two methods were significantly correlated (*p* < 0.0001) for both rivaroxaban (*r* = 0.98 95% CI [0.95–0.99]) and apixaban samples (*r* = 0.94 95% CI [0.89–0.97]). The dRVVT screen ratio increased in a concentration-dependent manner (*p* < 0.0001) in the presence of rivaroxaban and apixaban with a more pronounced effect of the former (*r* = 0.81 vs. *r* = 0.72, respectively) (Figures 2A,C). dRVVT screen ratios were elevated (i.e., ≥ 1.20) in 100% of rivaroxaban and 92% of apixaban samples (Table 2). Samples with elevated screen results were further tested using confirm assay. Screen/confirm ratios remained positive in 92 and 72% of the samples, respectively. SCT screen ratios were less correlated with rivaroxaban concentration (*p* < 0.0001, *r* = 0.71) compared to dRVVT screen ratios whereas they were not with apixaban concentration (*p* = 0.724) (Figures 2B,D). SCT screen ratios were elevated in 50% of rivaroxaban and 64% of apixaban samples. As for dRVVT assays, samples with elevated SCT screen results were further tested using the SCT confirm reagent. SCT screen/confirm ratios were positive in 28 and 41% of the samples, respectively (Table 2).

**Table 1:** FXa inhibitors concentrations in pre- and post-filtration samples.

	**Rivaroxaban samples**			**Apixaban samples**		
	**Pre-filtration**	**Post-filtration**	**% of neutralization**	**Pre-filtration**	**Post-filtration**	**% of neutralization**
Anti-Xa assay (ng/mL)	63 [26; 363]	<18 [<18; 22]		126 [33; 370]	<18	
HPLC-MS/MS (ng/mL)	64.8 [17.6; 311.4]	<2 [<2; 2.4]	100 [99.7; 100]	92.1 [37.1; 390.7]	<2 [<2; 9.6]	100 [97.4; 100]

*Median [min; max] values are reported. Rivaroxaban concentration was measured using specific anti-Xa assay or HPLC-MS/MS in 30 and 37 samples, respectively. Apixaban concentration was measured in 38 and 41 samples, respectively. Total neutralization (100%) corresponds to xaban concentration below the lower limit of quantification of HPLC-MS/MS (i.e., 2 ng/mL)*.

**Table 2:** Percentage of positive LA results in pre- and post-filtration patients' samples.

	**dRVVT**		**SCT**		**dRVVT and/or SCT**	
	**Screen ratio**	**Screen ratio/confirm ratio**	**Screen ratio**	**Screen ratio/confirm ratio**	**Screen ratio**	**Screen ratio/confirm ratio**
**Rivaroxaban**						
Pre-filtration	38/38 (100%)	35/38 (92%)	18/36 (50%)	10/36 (28%)	36/36 (100%)	33/36 (92%)
Post-filtration	6/38 (16%)	6/38 (16%)	3/36 (8%)	3/36 (8%)	7/36 (19%)	7/36 (19%)
**Apixaban**						
Pre-filtration	36/39 (92%)	28/39 (72%)	25/39 (64%)	16/39 (41%)	37/39 (95%)	30/39 (77%)
Post-filtration	18/39 (46%)	16/39 (41%)	10/39 (26%)	7/39 (18%)	22/39 (56%)	20/39 (51%)

*Positive result corresponds to a ratio ≥ 1.20. dRVVT, dilute Russell viper venom time; SCT, silica clotting time*.

### Effect of DOAC Filter on Direct Oral FXa Inhibitor Plasma Concentrations

Seventy-eight samples were analyzed using HPLC-MS/MS before and after treatment with DOAC Filter: 37 rivaroxaban and 41 apixaban samples. Sixty-eight were also analyzed using specific anti-Xa assays: 30 rivaroxaban and 38 apixaban samples. The remaining samples could not be tested with specific anti-Xa assays due to insufficient post-filtration sample volume. DOAC Filter significantly reduced the plasma concentrations of rivaroxaban (*p* < 0.0001) and apixaban (*p* < 0.0001) with an overall median % of neutralization of 100% (Table 1) as assessed with HPLC-MS/MS. Rivaroxaban concentration was below 2 ng/mL in 35 out of 37 post-filtration samples, the two remaining being equal to 2.35 and 2.43 ng/mL. Apixaban concentration was below 2 ng/mL in 24 out of the 41 post-filtration samples. A concentration-dependent adsorption effect was observed (*p* < 0.0001) with a Spearman correlation coefficient of *r* = 0.69 (95% CI [0.48–0.83]). The median residual apixaban concentration in the 17 remaining samples was 3.26 ng/mL [2.1–9.63]. Therefore, DOAC Filter substantially depleted patients' plasma samples from FXa inhibitor compounds.

### Effect of DOAC Filter on LA Testing in Samples From Patients Receiving Direct Oral FXa Inhibitors

**Figure 3:**
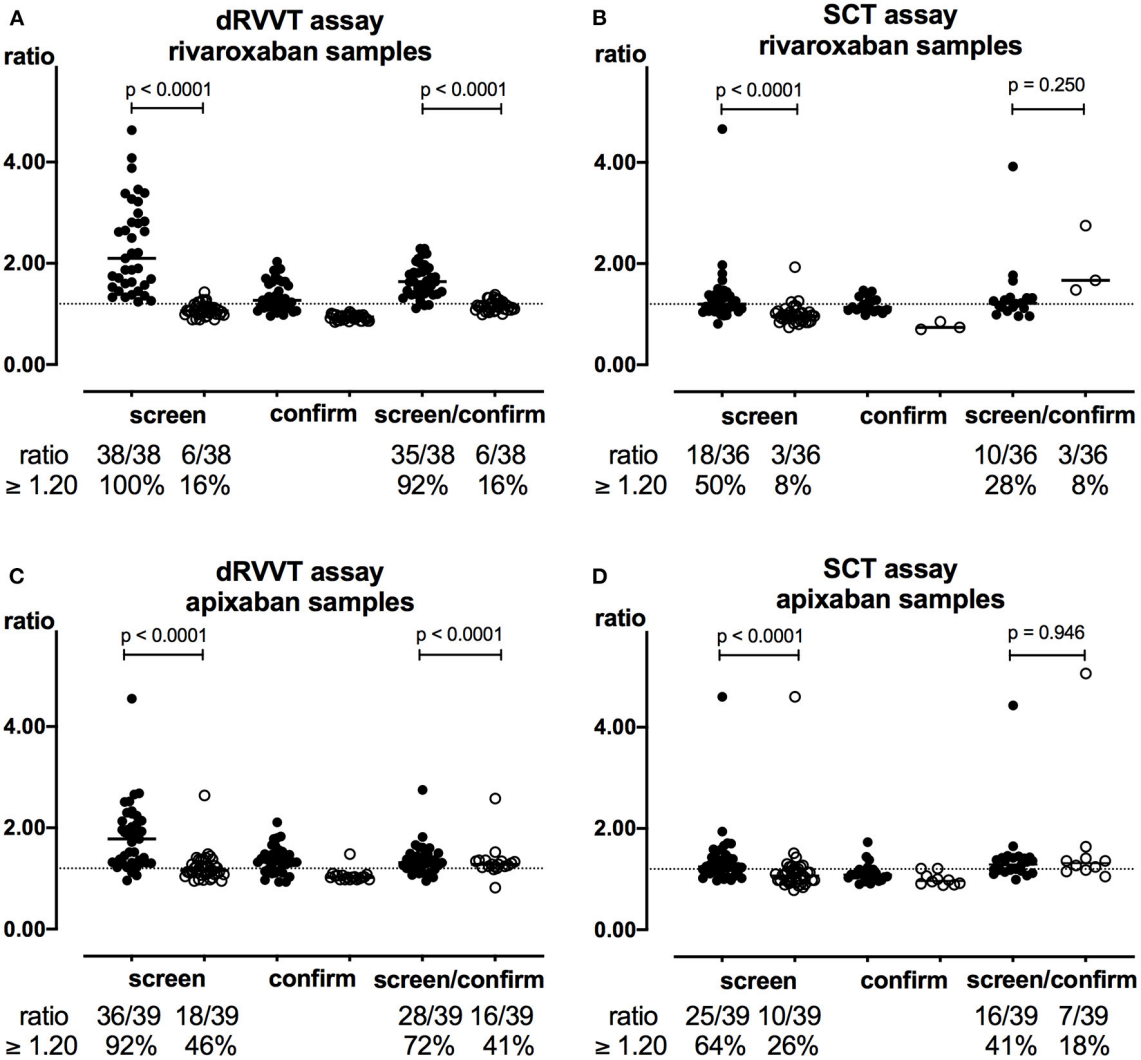
Effect of DOAC Filter on LA testing in FXa inhibitor samples. dRVVT **(A,C)** and SCT **(B,D)** screen, confirm, and screen/confirm ratios in rivaroxaban (**A,B**, *n* = 38 and *n* = 36, respectively) and apixaban (**C,D**, *n* = 39) samples were calculated before (closed symbols) and after (open symbols) treatment with DOAC Filter. Two rivaroxaban samples could not be tested with SCT assays due to insufficient sample volume. Only samples having elevated screen ratio (i.e., ≥ 1.20) were subsequently analyzed using confirm assays. Dashed line corresponds to the cutoff value of 1.20. Horizontal lines represent median values.

Following treatment with DOAC Filter, dRVVT and SCT screen and screen/confirm ratios significantly decreased in both rivaroxaban (Figures 3A,B) and apixaban samples (Figures 3C,D) along with the percentages of positive LA results (*p* < 0.0001). Drug interference with dRVVT and SCT screen was corrected in 84 and 83% of rivaroxaban and in 50 and 60% of apixaban pre-filtration samples having an elevated screen ratio. Following confirm assays in post-filtration samples, rivaroxaban interference with both tests was not changed whereas apixaban interference slightly decreased (Table 2). As LA testing is considered positive when at least one of the two tests is positive, DOAC Filter decreased positive LA results from 92 to 19% in rivaroxaban samples and from 77 to 51% in apixaban samples (Table 2). Plasma concentrations of FXa inhibitors in post-filtration samples with remaining positive LA results were <2 ng/mL [<2–2.43] for rivaroxaban (*n* = 7) and <2 ng/mL [<2–9.63] for apixaban (*n* = 20) samples as assessed by HPLC-MS/MS.

## Discussion

The present study provided evidence on the extent of interference of FXa inhibitors with LA testing and showed for the first time that DOAC Filter could remove, almost totally, these anticoagulant compounds from patients' plasma sample as assessed by the high-sensitivity and specific HPLC-MS/MS method, limiting therefore their confounding effect and allowing reliable LA detection in anticoagulated patients. Accurate LA testing may be useful in selected patients while they are still anticoagulated since it might affect the choice of the anticoagulant drug and the treatment duration (4, 5, 7).

Sevenet et al. have recently evaluated the capacity of DOAC Filter to remove direct oral anticoagulant compounds in normal pooled plasma spiked with increasing anticoagulant concentrations. They proved its neutrality with regard to dRVVT and LA-sensitive aPTT (PTT-LA) and also on a large panel of coagulation assays including prothrombin time, aPTT, thrombin time, fibrinogen, antithrombin, and protein C activities (29). Here, we showed for the first time that DOAC Filter might be a potential useful device in real-life clinical practice. It decreased the anticoagulant plasma concentration below the LLOQ of the commonly used specific anti-Xa assays in almost all the tested samples. FXa inhibitors' concentration got even below the LLOQ of HPLC-MS/MS as was observed with the vast majority of the rivaroxaban samples while a non-complete adsorption was observed in 17 out of 41 apixaban samples, which is consistent with the previous study performed with spiked samples (29). Therefore, DOAC Filter might have a better efficacy for rivaroxaban adsorption than for apixaban. This should be confirmed in a larger number of samples. Moreover, in post-filtration samples, LA results remained positive in 19% of rivaroxaban and 51% of apixaban samples despite an anticoagulant plasma concentration below 2 ng/mL in two-thirds of the cases. None of these rivaroxaban samples were tested positive for aCL or aβ_2_-GPI while three of the apixaban samples were tested positive for aCL and one for aβ_2_-GPI (being triple positive). Six out of the 20 LA-positive post-filtration apixaban samples had a FXa inhibitor concentration above 2 ng/mL (ranged between 2.10 and 9.63 ng/mL). To the best of our knowledge, no distinction between true and false LA-positive results is possible in this subgroup of samples. While Taipan snake venom time/ecarin clotting time would be useful for the detection of real LA-positive patients receiving FXa inhibitors, particularly in this subgroup of samples (31), these tests are not yet widely available in clinical practice. Although little known, the prevalence of aPL-positive results in patients with a history of thromboembolism was estimated to be around 10% (32, 33). Here, the % of positive LA results seemed well above, particularly in apixaban patients, even after exclusion of post-filtration samples with anticoagulant concentration remaining above 2 ng/mL. Of note, a repeat of this positive LA testing is required within a time interval of 12 weeks in order to establish the APS diagnosis.

Although the best option would be blood sampling for LA testing before starting any anticoagulant therapy, even when this is possible, most patients would be within the acute phase of thrombosis, making interpretation of results somewhat difficult (14). Usage of adsorbent devices based on activated charcoal as an additional tool for LA testing in treated patients has been recently suggested by the Scientific and Standardization Committee for lupus anticoagulant/antiphospholipid antibodies of the ISTH. However, a warning on the interpretation of the results was issued since complete reversal of the anti-FXa effect does not occur in every sample (14, 27, 34, 35). Moreover, adsorbent devices may interfere with clotting times, hence influencing the conclusion on LA testing as it was observed with DOAC Stop™ in some previous studies (28, 34, 36). In the present study, despite no significant difference observed in the results between pre- and post-filtration control and LA positive non-anticoagulated samples, some weakly elevated screen ratios turned out to be negative (i.e., <1.20) following treatment with DOAC Filter. It would thus be worthwhile to (i) evaluate whether DOAC Filter has any procoagulant effect using sensitive hemostasis assays such as thrombin generation test as was the case of DOAC Stop (36) and (ii) test a larger number of patients who are LA positive while receiving FXa inhibitors to see whether adsorbance of anticoagulant compounds yields consistent results. Moreover, if further studies show that treatment with DOAC Filter would change weak LA-positive samples devoid of any anticoagulant compound to LA negative, then DOAC Filter should only be used in plasma from FXa inhibitor-treated patients as was already shown with DOAC Stop™, which will be technically and economically wise in clinical practice. Indeed, in De Kesel's study, LA testing changed from positive to negative in 10 out of the 63 non-anticoagulant samples following treatment with DOAC Stop™ (35).

Plasma volume obtained post-filtration was reduced by around 30% resulting in a mean volume of 420 μL which could be limiting in clinical practice since this device cannot be used more than once. As such, two DOAC Filter cartridges might be necessary depending on the panel of coagulation assays to be performed on patient sample and requiring FXa inhibitors' quenching to avoid any misinterpretation.

Our study has some limitations. First, we evaluated the interference of FXa inhibitors with one specific test system for LA-sensitive aPTT (SCT) and dRVVT. Consequently, caution should be made on the generalization of our results to other reagents without local validation. Second, no known (real) positive LA samples from patients receiving direct FXa inhibitors could have been tested with DOAC Filter since these drugs are not prescribed in APS patients in accordance with the current recommendations (8–12). Therefore, we were unable to verify whether such samples would remain LA positive following filtration. Third, our study was not designed to determine the maximum rivaroxaban and apixaban levels that would be completely adsorbed by DOAC Filter. These remain to be established in appropriate future studies. Fourth, no mixing studies in a 1:1 proportion of tested samples with reference plasma were performed to rule out any potential coagulation factor deficiency since no correction of the dRVVT or aPTT-based assays in the presence of active FXa inhibitor molecules is expected as it was already proven by Merriman et al. (37). In case of doubt regarding any coagulation factor deficiency in xaban samples tested with dRVVT or SCT assays, levels of such factors should be specifically measured using appropriate dilutions of plasma to minimize the interference of FXa inhibitors.

In conclusion, DOAC Filter is a valuable new, ergonomic, and easy-to-use device allowing FXa inhibitors' *in vitro* removal from plasma samples, limiting therefore false LA-positive results in patients receiving FXa inhibitors.

## Data Availability Statement

The original contributions presented in the study are included in the article/Supplementary Material, further inquiries can be directed to the corresponding author/s.

## Ethics Statement

Ethical review and approval was not required for the study on human participants in accordance with the local legislation and institutional requirements. The patients/participants provided their written informed consent to participate in this study.

## Author Contributions

GJ conceived the study. CF, SE, and LG performed the research. CF and GJ analyzed the data and wrote the manuscript. XD performed the HPLC-MS/MS assays. SE, MS, JD, MF, VP, and XD critically read the manuscript and gave the final approval. All authors contributed to the article and approved the submitted version.

## Conflict of Interest

The authors declare that the research was conducted in the absence of any commercial or financial relationships that could be construed as a potential conflict of interest.
