# Supplementary material for: A Diagnostic Solution for Lupus Anticoagulant Testing in Patients Taking Direct Oral FXa Inhibitors Using DOAC Filter
Source: Front Med (Lausanne). 2021 May 31;8:683357. doi: 10.3389/fmed.2021.683357 (PMC8200390; doi:10.3389/fmed.2021.683357)
Supplement: Supplementary file 1 [file Table_1.DOCX]

**A diagnostic solution for Lupus Anticoagulant testing in patients taking direct oral FXa inhibitors using DOAC Filter**

Carine FARKH^1^, Syrine ELLOUZE^1^, Louis GOUNELLE^1^, Mama SAD HOUARI^1^, Jérôme DUCHEMIN^1^, Valérie PROULLE^1^, Michaela FONTENAY^1,2^, Xavier DELAVENNE^3,4^, Georges JOURDI^1,5,6,7^

**Supplementary Table I.** Discripant dRVVT and SCT pre- and post-filtration results in LA positive samples from non-anticoagulated patients

| **Samples #** | **dRVVT** | | | | **Samples #** | **SCT** | | | |
| --- | --- | --- | --- | --- | --- | --- | --- | --- | --- |
|  | screen ratio | | screen/confirm ratio | |  | screen ratio | | screen/confirm ratio | |
|  | pre-filtration | post-filtration | pre-filtration | post-filtration |  | pre-filtration | post-filtration | pre-filtration | post-filtration |
| 1 | 1.37 | 1.15 | 1.40 | 1.25 | a | 1.28 | 1.17 | 1.45 | 1.52 |
| 2 | 1.25 | 1.13 | 1.23 | 1.14 | b | 1.21 | 1.16 | 1.34 | 1.29 |
| 3 | 1.22 | 1.06 | 1.38 | 1.29 | c | 1.35 | 1.14 | 1.57 | 1.56 |
| 4 | 1.26 | 1.16 | 1.31 | 1.27 | d | 1.23 | 1.03 | 1.29 | 1.37 |
|  |  |  |  |  | e | 1.31 | 1.19 | 1.42 | 1.47 |

Positive result corresponds to a ratio > 1.20. dRVVT, dilute Russell viper venom time; SCT, silica clotting time
